# Analyzing Neck Circumference as an Indicator of CPAP Treatment Response in Obstructive Sleep Apnea with Network Medicine

**DOI:** 10.3390/diagnostics11010086

**Published:** 2021-01-07

**Authors:** Stefan Mihaicuta, Lucreţia Udrescu, Mihai Udrescu, Izabella-Anita Toth, Alexandru Topîrceanu, Roxana Pleavă, Carmen Ardelean

**Affiliations:** 1 Department of Pulmonology, “Victor Babeş” University of Medicine and Pharmacy Timişoara, 300041 Timişoara, Romania; stefan.mihaicuta@umft.ro (S.M.); toth_izabella87@yahoo.com (I.-A.T.); 2 CardioPrevent Foundation, 3 Calea Dorobanţilor, 300134 Timişoara, Romania; carmenardelean79@yahoo.com; 3 Department I—Drug Analysis, “Victor Babeş” University of Medicine and Pharmacy Timişoara, 2 Eftimie Murgu Sq., 300041 Timişoara, Romania; 4 Department of Computer and Information Technology, University Politehnica of Timişoara, 2 Vasile Pârvan Blvd., 300223 Timişoara, Romania; mihai.udrescu@cs.upt.ro (M.U.); alexandru.topirceanu@cs.upt.ro (A.T.); 5 Timişoara Institute of Complex Systems, 18 Vasile Lucaciu Str., 300044 Timişoara, Romania; 6 Department of Cardiology, “Victor Babeş” University of Medicine and Pharmacy Timişoara, 300041 Timişoara, Romania; roxana.pleava@gmail.com

**Keywords:** network medicine, obstructive sleep apnea syndrome, CPAP treatment response, anthropometric measures

## Abstract

We explored the relationship between obstructive sleep apnea (OSA) patients’ anthropometric measures and the CPAP treatment response. To that end, we processed three non-overlapping cohorts ($D_{1}$, $D_{2}$, $D_{3}$) with 1046 patients from four sleep laboratories in Western Romania, including 145 subjects ($D_{1}$) with one-night CPAP therapy. Using $D_{1}$ data, we created a CPAP-response network of patients, and found neck circumference (NC) as the most significant qualitative indicator for apnea–hypopnea index (AHI) improvement. We also investigated a quantitative NC cutoff value for OSA screening on cohorts $D_{2}$ (OSA-diagnosed) and $D_{3}$ (control), using the area under the curve. As such, we confirmed the correlation between NC and AHI (ρ=0.35, p<0.001) and showed that 71% of diagnosed male subjects had bigger NC values than subjects with no OSA (area under the curve is 0.71, with 95% CI 0.63–0.79, p<0.001); the optimal NC cutoff is 41 cm, with a sensitivity of 0.8099, a specificity of 0.5185, positive predicted value (PPV) = 0.9588, negative predicted value (NPV) = 0.1647, and positive likelihood ratio (LR+) = 1.68. Our NC =41 cm threshold classified the $D_{1}$ patients’ CPAP responses—measured as the difference in AHI prior to and after the one-night use of CPAP—with a sensitivity of 0.913 and a specificity of 0.859.

## 1. Introduction

Obstructive sleep apnea (OSA) is a chronic nocturnal disorder characterized by partial or complete episodes of upper airway collapse, which leads to oxygen desaturation and micro-arousals, causing symptoms, such as excessive sleepiness, fatigue and cognitive dysfunction [1]. OSA is recognized as an independent risk factor for several clinical severe conditions, such as systemic hypertension, arrhythmia, left ventricular dysfunction, abnormal glucose metabolism, coronary heart disease, stroke, and pulmonary hypertension [2]. Sleep apnea is further associated with higher mortality incidence caused by accidents [3], and cardiovascular diseases [4]; OSA may also represent a potential risk for cancer [5].

Studies show a prevalence of OSA in the general population of about 20%; the OSA diagnosis depends on the apnea–hypopnea index of higher than 5 in at least 1 hour of sleep [6]. The prevalence of moderate-to-severe sleep-disordered breathing (SBD) (≥15/h) in the Hypnolaus cohort was 23.4% in women and 49.7% in men [7]. The prevalence of OSA for subjects between 30 and 60 years is at 24% for men and 9% for women [8]. Sleep-disordered breathing has a significant prevalence in middle-aged subjects, with a decline after the age of 65 [9].

As neck thickness is an important indicator of OSA, the measurement of neck circumference (NC) has become standard practice in the current physical examination when there is suspicion of sleep apnea [10,11,12,13]. Additionally, epidemiological evidence indicates that an NC ≥43 cm is a better indicator of obstructive events frequency than body mass index (BMI) [11]. Other studies concluded that a big NC (adjusted to patient’s height) is considered as the most predictable clinical sign of OSA, coming close to 77% sensitivity and 82% specificity, and is the most significant factor that determines the clinical outcome of sleep apnea [14]. Overall, there is an almost consensus in previous research that NC is a reliable clinical indicator of OSA.

Many critical scientific problems can be modeled and visualized using complex networks [15]. For instance, biological and social patterns, the World Wide Web, metabolic networks, food webs, neural networks, pathological networks [16], and drug networks [17] are just a few real-world scientific and technological developments that we can use to uncover their properties [18]. The community structure of complex networks generally links to the behavior of the modeled system [17,19,20]. As such, our approach uses the analysis of the network topological communities in order to gain a better overview of the relationships between anthropometric risk factors in OSA [21]. Indeed, previous research using a network medicine approach acknowledged that NC is an essential objective parameter for OSA prediction scores [22].

Our main inspiration stemmed from the connections between OSA patient phenotypes and the various manifestations of this disease, found with complex network approaches [21,23]. In turn, this paper proposes a methodology that aims to identify specific patterns of response to CPAP treatment, taking into account the multiple connections between risk factors in a relevant patient population. Thus, by using tools put forward by network science, our work proposes a methodology that associates apnea risk groups with each CPAP treatment response pattern. Such analytical findings allowed us to find a link between the CPAP response prediction and NC through network medicine.

## 2. Materials and Methods

The study presented in this paper is based on the approval granted by The Ethical Committee of Victor Babeş Hospital, Timişoara, Romania (approval number 10/12.10.2013). As such, we develop a two-step approach. First, we apply network analysis on a relevant population of consecutive patients (database $D_{1}$, 145 patients with one-night CPAP therapy) and determine the relevant measures which associate with CPAP treatment response. Second, we statistically analyze a population of patients (supporting databases $D_{2}+D_{3}$ with 901 male patients) to fine-tune the analytically determined anthropometric measures. Supporting Information file Supplementary Material Datasets.xlsx includes databases $D_{1}$, $D_{2}$, and $D_{3}$.

Throughout this study, we refer to different categories of OSA severity based on patients AHI. The apnea–hypopnea index is the principal measure in polysomnography and represents the average number of apneas and hypopneas per hour of sleep. The AHI value classifies each patient in one of the following severity categories [24]: normal (or low-risk) apnea (norm) for AHI <5, mild sleep apnea (mild) for 5≤ AHI <15, moderate sleep apnea (mod) for 15≤ AHI <30, and severe sleep apnea (sev) when AHI ≥30.

### 2.1. Subjects

In this retrospective study, we define cohort $D_{1}$ consisting of 145 new OSA patients (age range 8–84 years, fully evaluated for OSA diagnosis) who were subject—for the first time—to a one night CPAP treatment, with a duration of 4–5 h/night (4.4 h on average); $D_{1}$ is the result of an APAP titration where the optimal pressure was 9.1 cmH_2_O.

The supporting cohorts $D_{2}+D_{3}$ consist of 901 consecutive adult male subjects (≥18 years old at the time of evaluation). All subjects in this study were diagnosed in 4 sleep centers from the Timisoara area, Western Romania, and were referred to sleep laboratories for OSA evaluation from June 2012 to April 2018. Our database $D_{2}$ includes the 836 patients with a complete evaluation that suggests OSA and excludes predominantly central apnea (≥10 central events/h); database $D_{3}$ includes the remaining 65 patients not diagnosed with OSA.

**Figure 1:**
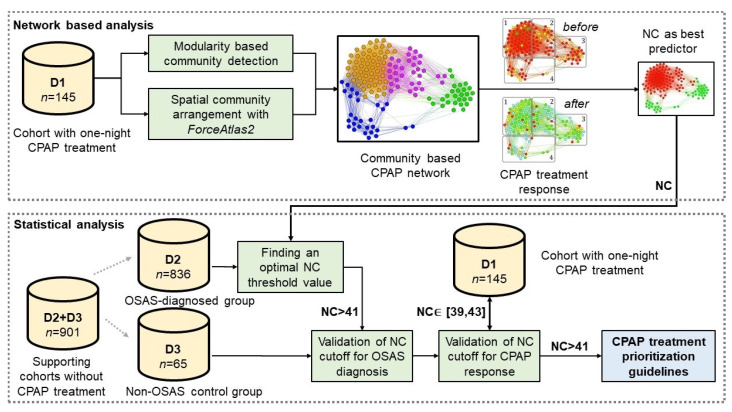
Overview of datasets and study design. The network-based analysis stage used cohort $D_{1}$ to model a CPAP patient network. By corroborating the community structure of this network with the CPAP treatment response of each patient (i.e., measured as AHI improvement), we extracted neck circumference (NC) as the most significant indicator of AHI improvement. We further used this information in the statistical analysis stage, in which used a larger $D_{2}+D_{3}$ supporting cohort to find an optimal NC threshold value for OSA-diagnosed patients. Cohort $D_{3}$ was the non-OSA control group. The study resulted in the definition of a rule of thumb guideline for CPAP treatment prioritization of patients with OSA (blue).

Table 1 presents all anthropometric patient data and clinical parameters of the study cohorts. Our $D_{1}$ and $D_{2}+D_{3}$ datasets are not overlapping, as presented in the study and database overview from Figure 1.

### 2.2. Data Collection

We obtained written, informed consent from each patient. At the initial visit, we gathered patient data consisting of demographics, medical history, and anthropometrics (including NC). We performed an overnight polysomnography following guidelines and recommendations [25]. As such, we recorded electroencephalogram, electrocardiogram, submental electro-myogram and electrooculogram, oximetry; at the same time, we performed airflow measurement, using both nasal pressure transducer and oronasal thermistor. The interpretation of the signals conforms to the standard criteria [25].

### 2.3. Network Analysis

In this paper, we follow the network medicine approach [15,26], which can uncover complex phenotype relationships in respiratory medicine [21,23,27,28]. Using the data from $D_{1}$, we construct a complex network G=(V,E), with *V* the node (or vertex) set and *E* the link (or edge) set. Each patient is represented by a node $v_{i}\in V$; a link $e_{ij}$ between two nodes ($v_{i},v_{j}\in V$) exists if there is a *risk compatibility* relationship between the two corresponding patients. The risk compatibility exists if the two connected nodes—representing patients—fall within at least 4 out of 6 identical parameter classes:

- Gender (male or female),
- Age group (group 0: ≤20 yrs; group 1: 20–40 yrs; group 2: 40–60 yrs; group 3: >60 yrs),
- Blood pressure BP (LBP or HBP, based on systolic BP >140 or diastolic BP >90 for HBP),
- Obesity (not obese or obese, based on BMI > 30 for obese),
- Neck circumference (thin neck tN or thick neck TN, based on NC ≥ 40 cm for women, ≥43 cm for men for TN—meaning that tN and TN are complementary Boolean variables; when tN = 0, then TN = 1 and vice-versa),
- Epworth sleepiness score (without sleepiness or with sleepiness, based on ESS ≥ 11 for sleepiness).

In Figure 1 we offer an intuitive representation comprising the usage of all three datasets, and the design of our retrospective study.

We select these six specific input parameters according to the state of the art OSA research, and their cutoff points are based on the clinical practice of our sleep centers [21,29,30], which follow international guidelines [14,31,32].

Based on the enumerated cutoff values, we classify the input parameters into 2 classes (4 classes for age) instead of using them quantitatively in order to be able to define explicit patient compatibility. Accordingly, this means that if two patients are in the same parameter class, they have a risk compatibility; conversely, different classes suggest no risk compatibility. Consequently, our network edge weight calculation between any two patients results in counting the number of compatible input parameter classes. All input parameters have equal weight in the edge calculation, and there is no critical attribute considered in network formation.

We used Gephi 0.8.1 [33] to generate the graphical representation, thus allowing us to extract the most critical attributes of the network, and to reveal the compatibility clusters. A compatibility cluster uniquely defines a specific OSA patient phenotype.

#### 2.3.1. Community Detection Algorithms

The network community detection techniques we used are modularity [34] and Force Atlas 2 layout algorithms [35], and combined in order to assess the correlation between patients response to CPAP treatment (i.e., categorized through direct AHI measurements available in D1) and their measured parameters linked to OSA (which determine the network topology and its community structure).

In a complex network G=(V,E), a clustering or community detection algorithm is an assignment $A_{m}$ of each node $v_{j}$ in one of the clusters $C_{i}$, with $\cup_{i=1}^{m}C_{i}=V$. As such, when modularity determines the assignment of nodes to their corresponding clusters $A_{m}=\left\{C_{1},C_{2},...,C_{m}\right\}$, the algorithm maximizes the modularity of clustering $A_{m}$ as follows:

(1) $$M_{A_{m}}=\sum_{C\in A_{m}}\left(\frac{|E_{C_{i}}|}{\left|E\right|}-\frac{\frac{1}{2}k_{C_{i}}^{2}}{\frac{1}{2}k^{2}}\right)$$

where |E| is the total number of edges in the network *G*, $|E_{C_{i}}|$ is the number of edges in cluster $C_{i}$, *k* is the total (i.e., accumulated) degree of vertices in *G*, and $k_{C_{i}}$ is the accumulated degree of vertices in cluster $C_{i}$. Therefore, the term $|E_{C_{i}}\left|/|E\right|$ represents the edge density of cluster $C_{i}$ relative to the density of the entire network, and the term $\frac{1}{2}k_{C_{i}}^{2}/\frac{1}{2}k^{2}$ represents the expected such relative density of $C_{i}$ [20].

A network layout algorithm assigns each vertex $v_{i}\in V$ a coordinate in a 2D space $\delta_{i}=\left(x_{i},y_{i}\right)\in R^{2}$. As such, each edge will have a length given by the Euclidean distance $\delta_{i,j}=\left|\delta_{i}-\delta_{j}\right|$. A force-directed or energy-based layout generates the $\delta_{i}$ for each $v_{i}$ using a dynamic, emergent process, where any two adjacent nodes $v_{i}$ and $v_{j}$ attract each other and any two non-adjacent nodes $v_{i}$ and $v_{k}$ repulse each other. We express such attraction/repulsion forces as $|\delta_{i}-\delta_{j}{|}^{\Phi }\cdot \vec{\delta_{i}\delta_{j}}$, where Φ=a for attraction, Φ=r for repulsion, and $\vec{\delta_{i}\delta_{j}}$ is the unit vector. The attraction between adjacent nodes decreases and the repulsion between non-adjacent nodes increases with the Euclidean distance between them; therefore, we have a≥0 and r≤0. In this paper, we use the Force Atlas 2 energy-based layout algorithm [35], which employs a dynamic complex process based on interacting attraction and repulsion forces to attain minimal energy in the layout:

(2) $$min\left\{\sum_{(V_{i},V_{j}),i\neq j}\left(\frac{|\delta_{i}-\delta_{j}{|}^{a}}{a+1}-\frac{|\delta_{i}-\delta_{j}{|}^{r}}{r+1}\right)\right\}$$

This way, the force-directed layouts generate topological clusters, as some specific network regions have higher than average edge densities. Noack [36] has demonstrated that modularity-based and force-directed layout communities/clusters are equivalent when a>−1 and r>−1, which, indeed, is the case for Force Atlas 2.

#### 2.3.2. Graph Modeling Based on Risk Compatibility

To obtain the CPAP patient network based on $D_{1}$, we filtered out all edges with weight <4 (i.e., patients having less than 4 out of 6 common parameter classes) to obtain the final CPAP patient network. Indeed, we can increase or decrease this threshold, but this process directly alters the density and number of communities obtained. For example, if the threshold is set to a low “1 out of 6,” then any two patients may be connected, resulting in a highly dense graph, and one single large community. Conversely, if the threshold is set to a very strict “6 out of 6” (i.e., two patients must be identical in terms of all six parameter classes), then the resulting graph is highly sparse, and has a large number of small non-representative communities. Using the “at least 4 out of 6” rule, we found the most representative network structure with a balanced number of communities.

**Figure 2:**
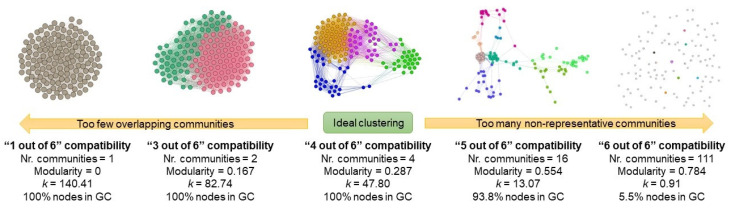
Ideal compatibility threshold of “at least 4 out of 6” common parameter classes used in the modeling of the CPAP patient network. If a lower threshold is used (i.e., less strict), too few, dense, and overlapping communities emerge. Conversely, if a higher threshold is used (i.e., more strict), too many non-representative communities emerge, and many nodes become completely disconnected from the giant component (GC) of the network.

The procedure of finding an optimal threshold is an empirical one, and is based on creating the graph, and then running modularity and Force Atlas 2 to quantify and visualize the obtained graph. We have previously applied a similar approach on OSA patients [21]; Figure 2 captures this exact process.

### 2.4. Statistical Analysis

We performed the Shapiro–Wilk test to examine the normal distribution of the variables of interest. As the continuous variables are not normally distributed, we analyze the correlations with Spearman’s rank test. The difference of continuous variables mean ranks was analyzed with the non-parametric Mann–Whitney U test. The results are summarized as median (interquartile range) for continuous variables and as percentages for categorical variables.

We used the receiver operating characteristic (ROC) curve to assess the classification ability of neck circumference for the OSA diagnosis [37]. The ROC plots the true-positive rate (sensitivity) against the false-positive rate (1-specificity) using a binary classifier that indicates OSA presence or absence. The area under the curve (AUC) derives from the ROC curve and represents the measure of NC discriminatory performance for OSA diagnosis. In other words, AUC represents the probability that the NC test applied over randomly selected patients in the given population will correctly classify them as having OSA or not. We calculated sensitivity, specificity, positive predicted value (PPV), and negative predicted value (NPV) for different cutoff values. We also report the positive likelihood ratio (LR+) and the negative likelihood ratio (LR-). The LR+ indicates how much the odds of OSA increase when the test is positive. Conversely, the LR- indicates how much the odds of the disease decrease when the test is negative.

We performed the statistical analysis with SPSS version 20.0 for Windows. A *p* value of less than 0.05 is considered statistically significant.

## 3. Results

### 3.1. Determining an Efficient Indicator

Our network community detection approach, applied in conformity with the state-of-the-art methodology [16,18,20], results in generating four distinct communities/clusters indicating how specific patterns of OSA are associated with the CPAP treatment response of patients. Based on the modularity and Force Atlas 2 algorithms, community structure is a well known emergent property of complex networks [17,38].

**Figure 3:**
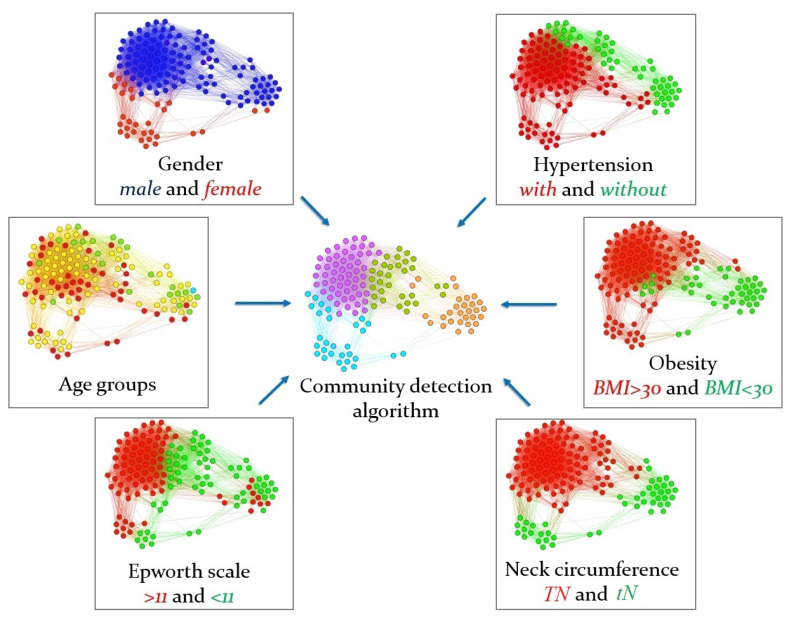
The network of 145 patients with overnight CPAP treatment shows the mapping of the six measurements (age, gender, blood pressure, BMI, Epworth scale, and neck circumference) that are relevant for the four patient communities detected for OSA (central panel). The four communities ($C_{1}$—magenta, $C_{2}$—olive, $C_{3}$—orange, $C_{4}$—cyan) emerged from the modeled risk compatibility between patients and were used to study the associations between patient risk factors and CPAP treatment response.

The CPAP patient network (n=145 patients) is depicted in the center of Figure 3, where we show the network with its distinctly colored communities (i.e., $C_{1}$—magenta, $C_{2}$—olive, $C_{3}$—orange, $C_{4}$—cyan), and around them, we present how each of the six measured criteria is associated with each cluster. Excepting the age group, all the other five measurements consistently associate with specific communities. We recorded AHI for all the patients in $D_{1}$ before and after the CPAP treatment to uncover a possible correlation between the four obtained communities and the effect of the CPAP treatment. Table 2 shows the percentage of patients in each of the four communities, based on the AHI severity class: *normal, mild, moderate, severe*. We measured AHI before and after the one-night treatment. The sizes of each community are: $C_{1}=55$ patients, $C_{2}=32$ patients, $C_{3}=29$ patients and $C_{4}=29$ patients.

**Figure 4:**
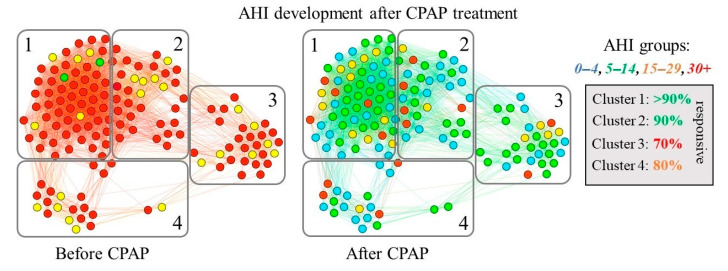
The network of 145 OSA patients highlighting the improvement of AHI in terms of severity class, after the over-night CPAP treatment.

Furthermore, in Figure 4, we highlight the AHI values for each patient before and after one night of CPAP treatment. Based on the classification using AHI, a patient may fall into one of four possible severity categories (i.e., *normal, mild, moderate, severe*). Then, we quantify the improvement of AHI in terms of severity class, after the over-night treatment.

We determined that communities $C_{1}$ and $C_{2}$ are the most representative in terms of patients’ AHI improvement. In Table 3 we show that patients belonging to $C_{1}$ and $C_{2}$ can be reclassified from severe to normal/mild OSA after CPAP treatment in a proportion of 87–89%. Conversely, the same proportions of AHI improvement in communities $C_{3}$ and $C_{4}$ are only within 68–77%. Thus, we consider membership to $C_{1}-C_{2}$ as a marker for a consistent CPAP treatment response. As such, we compared the characteristics of $C_{1}-C_{2}$ (best response class) with $C_{3}-C_{4}$ (good response class), and with the help of Table 4, we found that NC (thick neck) is the only parameter capable of classifying the two response classes. To summarize the comparison between the best and good response classes, we found that:

- Male gender is in both response classes, so it cannot be used as a classifier.
- HBP = 1 is inconsistent across the two response classes.
- Obese = 1 is in both response classes.
- TN = 1 (98–100%) is only in the best response class, and TN = 0 (3–38%) is only in the good response class.
- Sleepiness is inconsistent across the two response classes.

As such, a thick neck (TN, based on NC) is the best parameter for indicating the CPAP treatment response class. Corroborating Figure 3 and Figure 4, we notice that the two communities with ≈90% severity reduction (i.e., $C_{1}$, $C_{2}$) consist of male patients, who are also mostly obese. However, the same gender and obesity combinations also appear in communities 3 and 4. Therefore, neck circumference (NC) stands out as a significant indicator for efficient CPAP treatment response (see Table 4 for a detailed mapping of patient measurements on each community).

We further investigate the distribution of NC on a larger supporting dataset of male patients ($D_{2}$) and the correlation between NC and other OSA measurements. Thus, we target to obtain a certain NC threshold, above which we consider that CPAP treatment is effective.

### 3.2. Optimizing the Nc Threshold Value

Based on the conclusions drawn from $D_{1}$, namely, that NC is the main indicator of CPAP treatment response, we further use dataset $D_{2}$ to develop a statistical study on the NC cutoff point. Therefore, we do not claim any generalization of the subgroups in $D_{1}$, and $D_{2}$ is used solely for statistical analysis, while $D_{3}$ (non-OSA cohort) represents a control group for $D_{2}$ (OSA cohort).

The $D_{2}$ supporting cohort includes 836 male patients diagnosed with OSA after the polysomnographic evaluation. The age interval is 19–83 years (median 52, interquartile range 42–60), while the neck circumference ranges from 30 to 62 cm (median 45, interquartile range 42–47). Additionally, the median AHI is 38 events/h (interquartile range 22.8–57.7). There is a significant correlation between NC and AHI (ρ=0.35, p<0.001). The value of NC is significantly higher within the group of patients diagnosed with OSA $D_{2}$ than within the non-OSA patients’ group in $D_{3}$ (p<0.001).

**Figure 5:**
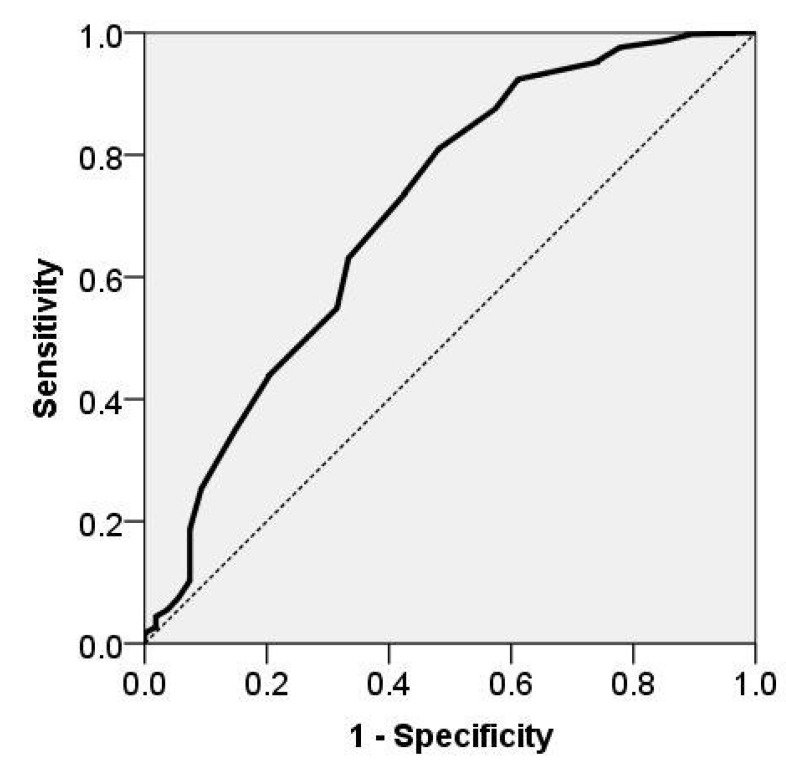
Receiver operator characteristic curve of neck circumference, for the differentiation between OSA and normal controls. The ROC curve illustrates the high OSA discriminatory performance of neck circumference—NC (area under curve AUC = 0.71, with a corresponding 95% confidence interval (CI) of 0.63–0.79, p<0.001).

The ROC curve analysis reveals the NC classification ability for the diagnosis of OSA (see Figure 5). The area under the ROC curve represents the percentage of patients within the OSA-diagnosed group $D_{2}$ that has a higher NC than the patients from the control group $D_{3}$. An area of 0.50 indicates a screening test that is no better than the chance of distinguishing the OSA diagnosed subjects’ group from the control group.

For our OSA study, the AUC is 0.71, as presented in Figure 5, which indicates that 71% of OSA diagnosed subjects have a higher NC than the subjects with no OSA. The obtained AUC value of 0.71 differs significantly (p<0.001) from the value corresponding to the null hypotheses (i.e., AUC = 0.50). Moreover, the value of 0.50 is not included within the 95% confidence interval of the AUC (0.63–0.79), suggesting that the discrimination capability of NC is statistically significant (Table 5).

The optimal NC value is 41, with a sensitivity of 0.8099 and specificity of 0.5185. We obtained the optimal neck circumference value according to the Youden index criteria, which targets the maximum sum between sensitivity and specificity. The positive likelihood ratio LR+ is 1.68, indicating that a male patient with a neck circumference over 41 cm is 1.68 times more likely to have OSA than a male patient having the NC value below this cutoff point. Moreover, the positive predictive value of this score is 0.9588, indicating that 95.88% of the patients with NC >41 cm have OSA.

We subsequently divided the patients into two groups: a group of patients with neck circumference below 41 cm, and a group of patients with a neck circumference above this cutoff point. Table 6 presents a comparison between the sleep characteristics of patients with NC ≤41 cm and patients with NC above this cutoff point.

All sleep characteristics are significantly worse within the group of patients with NC >41 cm, except for the minimum oxygen desaturation.

### 3.3. Evaluation of the Nc Threshold on the Cpap Patient Network

Finally, we return to the CPAP network based on $D_{1}$, and use the determined threshold of NC >41 to classify the patients based on their membership to communities C1–C4.

In the case of an ideal CPAP treatment response indicator (like NC >41), all patients in $D_{1}$ with NC >41 cm should belong to the *best* response class (communities C1–C2), while all other patients with NC ≤41 should belong to the *good* response class (communities C3–C4). Table 7 shows that our statistically derived indicator can classify patients with high precision. More precisely, 93.59% of patients with NC >41 are correctly assigned to the *best* response class, while 80.30% of patients with NC ≤41 are correctly assigned to the *good* response class. The statistical significance measurements for the *good* response class (NC ≤41) are sensitivity (TPR) of 0.913, specificity (TNR) of 0.859, respectively, for the *best* response class (NC >41) we measure sensitivity of 0.85 and specificity of 0.929. Moreover, Table 7 shows the classification performance of alternative NC thresholds, within 39–43 cm. Given these statistical results, we conclude that NC >41 is the ideal treatment response indicator.

## 4. Discussion

Neck circumference is often considered an independent risk factor used for the screening of OSA severity. Indeed, a large NC may be determined by obesity, genetics etc. [39], thus, further leading to the development of OSA. Nonetheless, we consider NC a useful *marker* [40,41] or indicator of CPAP treatment response and early OSA diagnosis.

In a study by Hoffstein et al. [42] on 670 patients obese subjects suspected of having OSA had a higher NC compared to equally obese non-apneic snorers, although their abdominal circumferences were similar. As such, this study suggests that the NC value can distinguish between sleep apnea and snoring [42]. Ahbab et al. identified NC as an independent risk factor for severe OSA [10]. The neck circumference was confirmed by [14] as a specific indicator of OSA, especially in patients with excess neck fat deposition in anterolateral to the upper airway [43]. Prior research also indicates that adults—especially males—with a large NC are more likely to develop OSA. Even for children, a big NC associates with an increased risk of OSA; however, this observation only holds for the male subjects [44].

Our research group reports previous studies using network science to identify subgroups (phenotypes) of patients with OSA [21,22,29]. However, in this retrospective study, we focus on using network analysis explicitly to analyze patients’ response to CPAP treatment. To this end, $D_{1}$ (n=145 patients) is the core dataset of our analysis, as it is the cohort of patients with one night CPAP treatment.

Our study confirms that NC is a reliable OSA indicator that reflects its severity. The positive correlation of NC with AHI (ρ=0.35, p<0.001) suggests the NC ability. Consequently, the mean NC value for the group of OSA-diagnosed patients is significantly higher than the corresponding mean NC value for the control group. This result confirms the investigations of Yildirim et al., who observed that NC is significantly higher in the OSA group as compared with the control group and that there is a significant positive correlation between AHI and NC (ρ=0.477, p<0.001) [45]. The receiver operator characteristic curve analysis of NC for the differentiation of OSA from normal controls reveals a good prediction accuracy (AUC = 0.71, 95% CI 0.63–0.79, p<0.001). In our study, the optimal cutoff point is 41 cm with a sensitivity of 0.8099 and specificity of 0.5185, which indicates that patients with an NC ≤41 cm are less likely to be diagnosed with OSA using polysomnography. Our result suggests that it is not necessary to have a very thick neck in order to develop OSA, even for male subjects [22,46].

Our study also emphasized the classification ability of neck circumference for CPAP responsiveness, in a population cohort of people referred to sleep labs for OSA evaluation and treatment. The network analysis discovered NC as the best marker correlating with CPAP treatment, and our statistical analysis confirmed a certain NC threshold for reliable treatment and prioritization. Moreover, we found that male patients with NC ≥41 cm should have a higher priority for the overnight sleep study and treatment. Measures of OSA severity, such as AHI alone, appear more weakly associated with CPAP adherence [47,48]. Other studies show that it is possible to predict the initial lowest effective pressure CPAP with sufficient accuracy, and the optimal set of predictors consisted of only three variables: AHI, BMI, and NC [49]. In our study, we show that NC and initial AHI are good indicators for CPAP response measured by a significant reduction in AHI (i.e., difference in AHI prior and after the one-night use of CPAP). Bridging over the two studies, we suggest there is a link between the optimal CPAP pressure and AHI reduction based on initial AHI and NC used in conjunction as input variables.

In line with this study, a future development for our statistical analysis could be to describe the optimal OSA risk thresholds that optimize trade-offs between true positives, true negatives, false positives and false negatives, through the use of a total cost function [50]. Additionally, we could define a complementary patient network leading to new insights, based on an alternative inference method which consists of the identification of a significant maximum mutual information (MI) network [51]; in this case, two patients are connected with each other if their shared MI value is maximal with respect to all other patients for at least for one of the two patients.

### Limitations of The Study

First, we discuss the size of dataset $D_{1}$ consisting of patients with one night CPAP treatment. While our core dataset comprised only 145 CPAP patients, OSA related studies on small cohorts are not uncommon. We found other published studies having similar-sized cohorts [52,53,54].

Second, the number of women in the study (*n* = 33 [22.75%] in $D_{1}$) may be considered unrepresentative. However, given the size of $D_{1}$, the female population is not unexpected, based on the gender distribution for OSA, where women are known to be less susceptible to the disease [55,56].

Third, our conclusions regarding CPAP treatment response do not include women. As discussed beforehand, we present a retrospective study based on a consecutive cohort rather than a randomized general population. As such, our network analysis does not render conclusive results regarding CPAP response in women; thus, we need to perform further investigations to clarify their membership to cluster $C_{3}$.

Fourth, we evaluate patients based only on one night CPAP treatment, instead of longer monitoring of AHI evolution. Nevertheless, one night CPAP titration is a standard procedure in sleep medicine and represents a good indicator of long term CPAP treatment response [57,58]. Additionally, because we used APAP titration, the CPAP response needs more time to onset; therefore, the overall percentage of fully treated patients we measure is lower than expected.

Finally, it is worth noting that, in general, when applying network analysis, it is non-trivial to offer accurate quantitative assessments, but more common to offer qualitative assessments. As such, our claim focusing on NC is subject to the inherent interpretability of the obtained CPAP network model; nevertheless, we follow-up this observation with the robust statistical analysis approach (ROC) to validate our claims.

## 5. Conclusions

The central question of this study was whether anthropomorphic variables can indicate the CPAP treatment response. Indeed, we reached this conclusion by finding with the help of network analysis that NC is the best qualitative indicator of treatment response. Furthermore, the NC cutoff value for OSA diagnosed patients, is in line with the optimal cutoff value for the CPAP response.

Neck circumference is a reliable risk marker for patients suspected of OSA. Moreover, our novel network medicine interdisciplinary approach has uncovered that an NC >41 cm is a reliable and easy to measure indicator for efficient CPAP treatment.

To obtain an effective OSA prognosis, we can use NC in combination with other parameters or descriptors such as symptoms, age, gender, and BMI. To that end, we applied the determined NC cutoff point on already defined OSA phenotypes [21,22] to narrow down the target population even further. Nevertheless, in prioritizing male patients for CPAP treatment, we suggest as a rule of thumb that patients with NC higher than 41 cm will have better responses.

## Figures and Tables

**Table 1:** Anthropometric patient data and clinical parameters of the study cohorts: CPAP treatment group $D_{1}$ (n=145), OSA-diagnosed group $D_{2}$ (n=836), and non-OSA control group $D_{3}$ (n=65). We provide the results as either average ±SD or number and percentage *n* (%*n*). Patients in $D_{2}$ and $D_{3}$ did not undergo CPAP treatment.

Parameter	*D* ${}_{1}$	*D* ${}_{2}$	*D* ${}_{3}$
	n=145	n=836	n=65
Age (years)	52.79±12.32	51.75±12.47	43.63±18.66
Gender (male)	112 (77.24%)	836 (100.0%)	65 (100.0%)
Body-mass index (kg/m${}^{2}$)	33.17±6.64	33.13±6.37	27.81±6.37
Obesity (BMI > 30)	103 (71.03%)	522 (62.44%)	20 (30.77%)
Neck circumference (cm)	43.12±5.06	44.91±4.45	40.67±5.77
Thick neck (NC ≥ 43(M), ≥ 40 (F))	62 (42.76%)	489 (58.49%)	18 (27.69%)
Epworth sleepiness score (0–24)	11.81±4.98	10.01±5.07	6.73±5.06
Sleepiness (ESS ≥ 11)	84 (57.93%)	509 (60.89%)	20 (30.77%)
Mean AHI before CPAP	52.28±23.58	41.68±24.07	6.01±2.42
Severe OSA prevalence (AHI ≥ 30/h) before CPAP	142 (97.93%)	778 (93.06%)	0 (0%)
Mean AHI after CPAP	13.26±16.91	–	–
Severe OSA prevalence (AHI ≥ 30/h) after CPAP	55 (37.93%)	–	–

**Table 2:** Percentages of patients from each community ($C_{1}-C_{4}$) categorized into each of the four OSA severity classes (*norm, mild, mod, sev*) before and after one-night CPAP treatment.

Before CPAP					After CPAP			
	*norm*	*mild*	*mod*	*sev*	*norm*	*mild*	*mod*	*sev*
$C_{1}$	1.82	3.64	5.45	89.09	25.45	43.64	21.82	9.09
$C_{2}$	3.12	0	18.75	78.12	43.75	43.75	3.12	9.38
$C_{3}$	0	0	24.14	75.86	31.03	44.83	13.79	10.34
$C_{4}$	0	0	24.14	75.86	55.17	24.14	3.45	17.24

**Table 3:** Patient input parameter distribution for each community $C_{1}-C_{4}$. A value of 1 means that the parameter class is representative for the community, 0 means the inverse class is representative (e.g., HBP vs. LBP), and “-” means that none of the parameter classes is representative. The apnea–hypopnea index (AHI) improvement quantifies the percentage of patients who have reduced their OSA severity from sev to mild or norm after CPAP treatment.

	Gender	HBP	Obese	TN	Sleepiness	AHI Improvement
$C_{1}$	M (100%)	1 (84%)	1 (91%)	1 (98%)	1 (100%)	89.29%
$C_{2}$	M (94%)	−(59%)	1 (72%)	1 (100%)	0 (0%)	87.50%
$C_{3}$	M (93%)	0 (10%)	0 (10%)	0 (3%)	−(31%)	68.75%
$C_{4}$	F (100%)	1 (93%)	1 (93%)	−(38%)	−(59%)	77.78%

**Table 4:** Anthropometric measurements for each of the four communities $C_{1}$–$C_{4}$ classified in each corresponding binary category. The values indicate the percentages of the patients from each community.

	Gender		BP		BMI		Neck		Sleepiness		Age Group			
	M	F	Low	High	Normal	Obese	Thin	Thick	No	Yes	<20	20–40	40–60	>60
$C_{1}$	100	–	16.36	83.64	9.09	90.91	1.82	98.18	0	100	–	12.73	54.55	32.73
$C_{2}$	93.75	6.25	40.62	59.38	28.12	71.88	–	100	100	–	–	25	43.75	31.25
$C_{3}$	93.1	6.9	79.31	20.69	89.66	10.34	96.55	3.45	58.62	31.38	3.45	24.14	51.72	20.69
$C_{4}$	–	100	6.9	93.1	6.9	93.1	62.07	37.93	41.38	58.62	–	–	62.07	37.93

**Table 5:** ROC analysis for the discrimination between OSA and normal control subjects, based on neck circumference. ROC = receiver operator characteristic, PPV = positive predicted value, NPV = negative predicted value, LR+ = positive likelihood ratio, LR− = negative likelihood ratio.

Cutoff Points	Sensitivity	Specificity	PPV	NPV	LR+	LR−
39	0.9237	0.3889	0.9544	0.2692	1.51	0.19
40	0.8755	0.4259	0.9547	0.1983	1.53	0.29
41	0.8099	0.5185	0.9588	0.1647	1.68	0.37
42	0.7376	0.5741	0.9599	0.1366	1.73	0.46
43	0.6305	0.6667	0.9632	0.1154	1.89	0.55
44	0.5489	0.6852	0.9602	0.0989	1.74	0.66
45	0.4391	0.7963	0.9676	0.0931	2.16	0.70
46	0.3494	0.8519	0.9703	0.0865	2.36	0.76

**Table 6:** Comparison between sleep characteristics of patients with neck circumference NC ≤41 cm and patients with NC above this cutoff point; the p-values were obtained with the Mann–Whitney U tests and are presented as median (1st quartile–3rd quartile; quartiles obtained with Turkey’s method). Q1 = 1st quartile, Q3 = 3rd quartile, MAD = mean apnea duration, AHI = apnea–hypopnea index, Obstructive MAD = Obstructive mean apnea duration, SpO2min = minimum oxygen desaturation.

Sleep Characteristics	NC ≤41	NC >41	*p*-Value
	(*n* = 170)	(*n* = 631)	
	Median (Q1–Q3)	Median (Q1–Q3)	
MAD	20.5 (18.6–22.6)	21.2 (19–24)	0.003
Obstructive MAD	17.7 (15.1–21.1)	19.3 (16.5–23.2)	<0.001
SpO2min	86 (81–89)	80 (70–86)	<0.001
Desaturation index (events/h)	6 (2.9–15.5)	23 (8.3–51.8)	<0.001
AHI	23.3 (12.8–40.3)	41.4 (26.5–63.2)	<0.001
Obstructive events	6.5 (2.6–19)	16.9 (6.7–37.6)	<0.001

**Table 7:** Classification of patients from $D_{1}$ into communities $C_{1}$–$C_{4}$ based on the NC threshold values within range 39–43 cm. We provide the corresponding statistical tests for each of the two response classes, good and best.

Response Class	Total	$C_{1}$	$C_{2}$	$C_{3}$	$C_{4}$	TPR	TNR	PPV	NPV
NC ≤ 39 (*good*)	29	0 (0%)	0 (0%)	11 (37.93%)	18 (62.07%)	0.500	1.000	1.000	0.800
NC>39 (*best*)	116	55 (47.41%)	32 (27.58%)	18 (15.51%)	11 (9.48%)	1.000	0.500	0.750	1.000
NC ≤ 40 (*good*)	42	0 (0%)	1 (2.38%)	19 (45.23%)	22 (52.38%)	0.706	0.990	0.976	0.858
NC>40 (*best*)	103	55 (53.40%)	31 (30.10%)	10 (9.70%)	7 (6.80%)	0.988	0.711	0.834	0.976
NC ≤ 41 (*good*)	53	1 (1.88%)	1 (1.88%)	28 (52.83%)	23 (43.39%)	0.879	0.978	0.962	0.929
NC>41 (*best*)	92	54 (58.69%)	31 (33.69%)	1 (1.09%)	6 (6.52%)	0.977	0.883	0.924	0.963
NC ≤ 42 (*good*)	66	6 (9.09%)	7 (10.61%)	25 (37.88%)	28 (42.42%)	0.913	0.859	0.803	0.940
NC>42 (*best*)	79	49 (62.02%)	25 (31.64%)	1 (1.26%)	4 (5.06%)	0.850	0.929	0.936	0.835
NC ≤ 43 (*good*)	83	15 (18.07%)	14 (16.86%)	26 (31.32%)	28 (33.73%)	0.931	0.681	0.650	0.939
NC>43 (*best*)	62	40 (64.51%)	18 (29.03%)	0 (0%)	4 (6.45%)	0.667	0.954	0.935	0.741

## Data Availability

The authors confirm that the data supporting the findings of this study are available within the article and its supplementary material.

## Supplementary Materials

The following is available at https://www.mdpi.com/2075-4418/11/1/86/s1, Datasets.xlsx.

## Abbreviations

The following abbreviations are used in this manuscript:

OSA	Obstructive Sleep Apnea
CPAP	Continuous Positive Airway Pressure
AHI	Apnea-Hypopnea Index
SDB	Sleep-Disordered Breathing
NC	Neck Circumference
BMI	Body-Mass Index
HBP	High Blood Pressure
LBP	Low Blood Pressure
tN	Thin Neck
TN	Thick Neck
ROC	Receiver Operating Characteristic
AUC	Area Under the Curve
PPV	Positive Predicted Value
NPV	Negative Predicted Value
TPR	True Positive Rate
TNR	True Negative Rate
